# Residual NADPH Oxidase Activity and Isolated Lung Involvement in X-Linked Chronic Granulomatous Disease

**DOI:** 10.1155/2012/974561

**Published:** 2012-11-05

**Authors:** Maria J. Gutierrez, George D. McSherry, Faoud T. Ishmael, Alexandra A. Horwitz, Gustavo Nino

**Affiliations:** ^1^Division of Pulmonary, Allergy and Critical Care Medicine, Department of Medicine, Pennsylvania State University College of Medicine, Hershey, PA 17033, USA; ^2^Divisions of Pediatric Allergy and Immunology and Pediatric Rheumatology, Department of Pediatrics, Pennsylvania State University College of Medicine, Hershey, PA 17033, USA; ^3^Division of Pediatric Infectious Diseases, Department of Pediatrics, Pennsylvania State University College of Medicine, Hershey, PA 17033, USA; ^4^Department of Biochemistry and Molecular Biology, Pennsylvania State University College of Medicine, Hershey, PA 17033, USA; ^5^Divisions of Pediatric Pulmonary and Pediatric Sleep Medicine, Department of Pediatrics, Penn State Hershey Children's Hospital, Pennsylvania State University College of Medicine, 500 University Drive, Hershey, PA 17033-0850, USA

## Abstract

Chronic granulomatous disease (CGD) is characterized by inherited immune defects resulting from mutations in the NADPH oxidase complex genes. The X-linked type of CGD is caused by defects in the CYBB gene that encodes gp91-phox, a fundamental component of the NADPH oxidase complex. This mutation originates the most common and severe form of CGD, which typically has absence of NADPH oxidase function and aggressive multisystemic infections. We present the case of a 9-year-old child with a rare CYBB mutation that preserves some NADPH oxidase activity, resulting in an atypical mild form of X-linked CGD with isolated lung involvement. Although the clinical picture and partially preserved oxidase function suggested an autosomal recessive form of CGD, genetic testing demonstrated a mutation in the exon 3 of CYBB gene (c.252 G>A, p.Ala84Ala), an uncommon X-linked CGD variant that affects splicing. Atypical presentation and diagnostic difficulties are discussed. This case highlights that the diagnosis of mild forms of X-linked CGD caused by rare CYBB mutations and partially preserved NADPH function should be considered early in the evaluation of atypical and recurrent lung infections.

## 1. Introduction

Chronic granulomatous disease (CGD) is one of the most prevalent primary immunodeficiency syndromes with an incidence of 1 case per 250,000 live births in the United States [1, 2]. CGD is characterized by inherited defects in the innate immune system resulting from mutations in the genes encoding any of the five components of the nicotinamide adenine dinucleotide phosphate (NADPH) oxidase complex, including gp91-phox, p22-phox, p40-phox, p47-phox, and p67-phox [1, 2]. As a result, phagocytes fail to generate superoxide anion and related phagocyte-derived reactive oxygen intermediates [1, 2]. The latter immune defect causes susceptibility to recurrent life-threatening fungal and bacterial infections, dysregulated granulomatous inflammation, and autoimmunity [1–4]. In about two-thirds of the cases, CGD is an X-linked disorder caused by mutations in the cytochrome b-245 beta (CYBB) gene, which maps at Xp21.1 [1–5]. CYBB mutations lead to defects in the membrane-bound gp91-phox protein, a fundamental component of the NADPH oxidase complex [5]. Patients who are gp91-phox deficient have largely suppressed NADPH function [6–8], resulting in severe multisystemic infections presenting in early life, with a mean age of 3 years at diagnosis, and an overall mortality rate close to 21% [1, 6–8]. In contrast, the autosomal recessive forms caused by defects of the p22-phox, p47-phox, p67-phox, and p40-phox components of the NADPH complex have residual NADPH oxidase function, with consequent less aggressive disease and longer survival rates [9–11].

We describe the case of a 9-year-old boy with a history of recurrent and slow to resolve episodes of pneumonia since 3 years of age. He was an otherwise healthy boy with normal growth and development. Dihydrorhodamine-123 (DHR) testing identified an abnormal neutrophil oxidative burst but residual NADPH oxidase function. His isolated lung involvement and partially preserved oxidase function suggested an autosomal recessive form of CGD. Nonetheless, genetic testing demonstrated a mutation in the exon 3 of CYBB gene (c.252 G>A, p.Ala84Ala), a very rare X-linked CGD variant that affects splicing [12]. This instructive case illustrates that CGD is an inherited disease with a heterogeneous phenotype more closely correlated with residual NADPH oxidase activity than with genetic testing results alone. Given that CYBB mutations are typically linked to severe CGD and a virtually absent oxidative burst [6–8], the residual oxidase activity present in this case provides novel insights into the complex genetic and posttranslational mechanisms involved in the control of NADPH oxidase function.

## 2. Case Report

At the age of 9.4 years, a US born Hispanic boy, child of nonconsanguineous parents, was admitted to our tertiary care children's hospital for evaluation of recurrent pneumonia. The patient had a history of five episodes of chest-X-ray-documented pneumonia beginning at the age of three years. Most of the episodes were remarkable for the need of in-hospital intravenous antibiotics due to persistent cough and fever. He was otherwise a normally active third grader when not suffering from pneumonia. He had been born at term after an uncomplicated pregnancy. He had no history of recurrent sinusitis, otitis, cellulitis, lymphadenitis, osteomyelitis, sepsis, meningitis, deep organ abscesses, or poor healing. He lived at home with his parents and a younger sister. There were no family members with recurrent infections or pneumonia. Previous workup for recurrent pneumonia had included two bronchoscopies with bronchoalveolar lavage (BAL) that revealed no specific respiratory pathogens or anatomical abnormalities. Immunologic evaluations had shown elevated IgG (1728 mg/dL) and IgA (408 mg/dL) levels, protective antitetanus titers, and normal pneumococcal antibody response. No lymphocyte subsets or evaluation of phagocytic function had been obtained. Tuberculosis, histoplasmosis, human immunodeficiency virus (HIV) infection, Wegener's granulomatosis, sarcoidosis, and cystic fibrosis had all been excluded prior to this admission.

**Figure 1:**
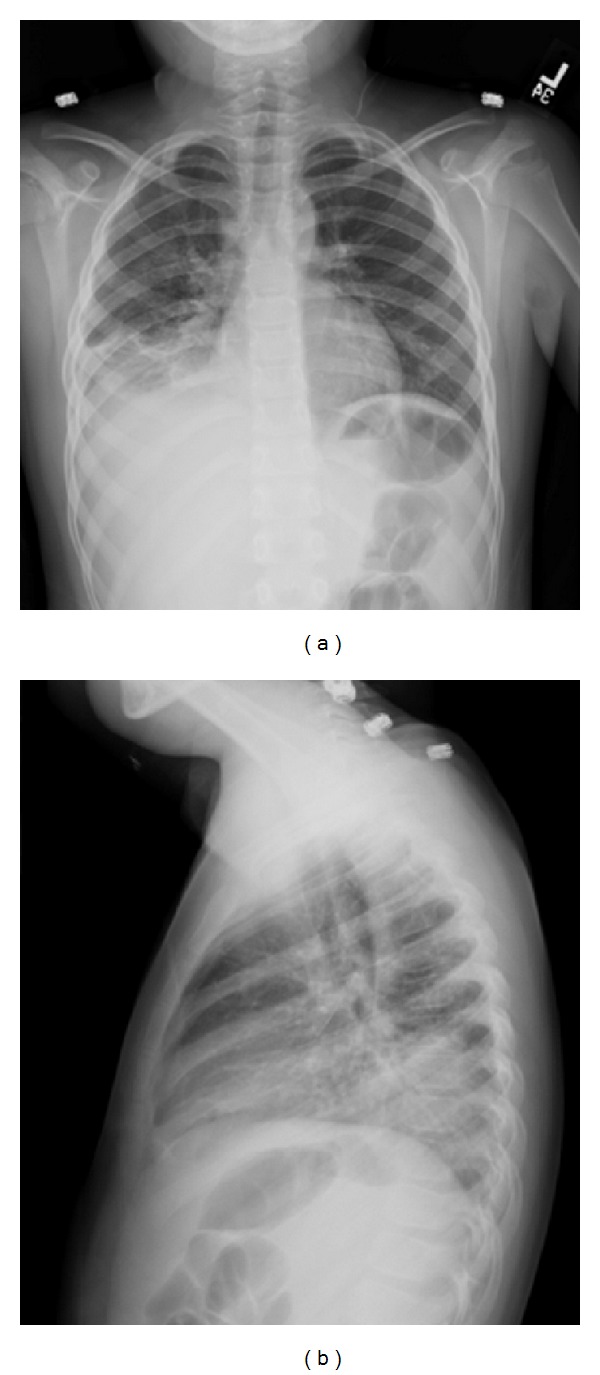
Chest radiographs during admission. Images demonstrating right lower lobe infiltrates. These films were taken at 9 years of age.

On examination, he was a well-developed boy (weight between 75th–90th percentiles) with fever (40°C) and tachypnea but without respiratory distress or hypoxemia. Crackles and tubular sounds were heard over the right lung. A chest radiograph (CXR) demonstrated right-sided pulmonary infiltrates (Figure 1). Complete blood count (CBC) revealed mild leukocytosis (14,000 cells per mm^3^) with neutrophilia (74%). Intravenous ampicillin-sulbactam was initially started but it was changed to vancomycin and ceftriaxone due to persistent cough and fever. A bronchoscopy revealed positive *Aspergillus* antigen (1.5. titers) in the BAL fluid, and respiratory culture yielded >100,000 colonies/mL of *Streptococcus viridans*. Pneumocystis direct fluorescent antibody (DFA), acid-fast bacillus (AFB) staining and cultures for *Nocardia*, *Mycobacterium tuberculosis*, fungi, and viruses were all negative.

**Figure 2:**
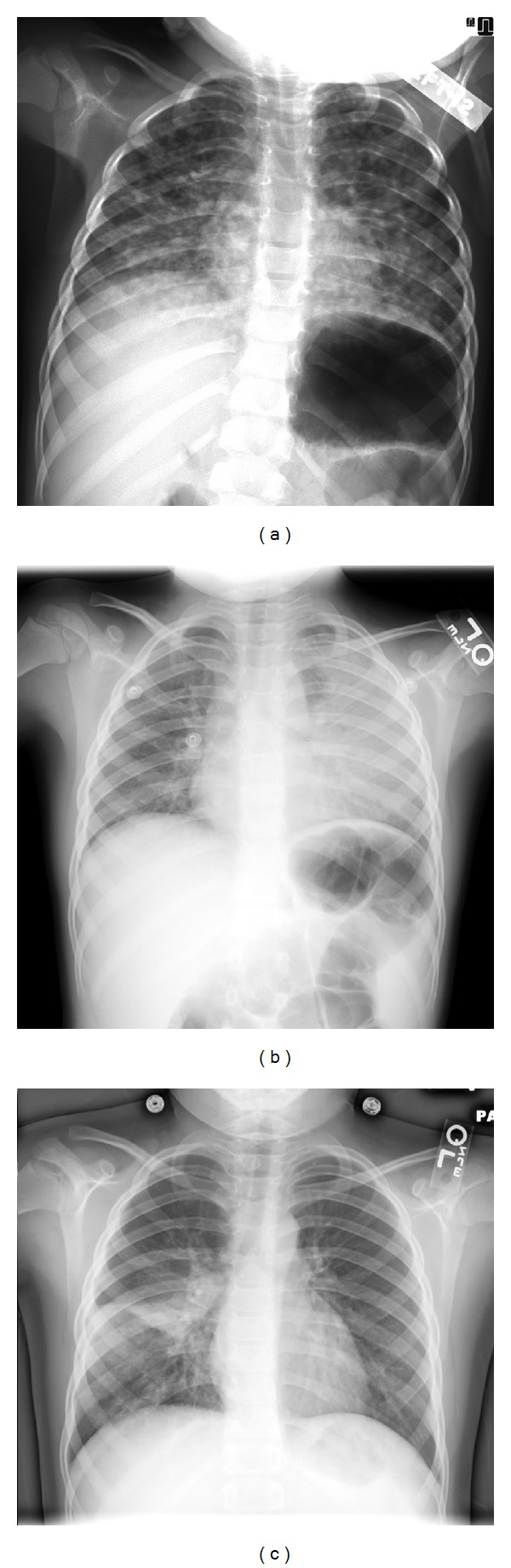
Chest radiographs prior to admission. Images demonstrating focal opacities with variable patterns, from extensive diffuse nodularity (a) to large opacifications involving several areas of the lungs ((b) and (c)). These films were taken at 3 years (a), 6 years (b), and 8 years (c) of age during prior episodes of pneumonia.

**Figure 3:**
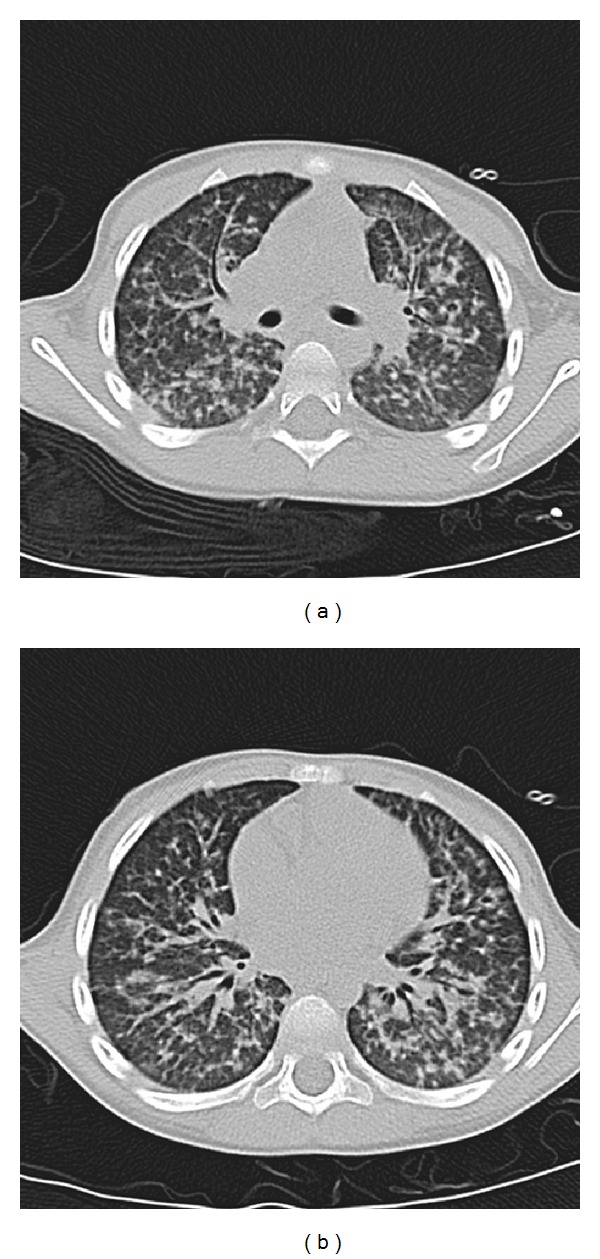
Chest CT prior to admission. Imaging showing diffuse nodular pattern involving different areas of the lungs. There is absence of air trapping or bronchiectatic changes usually seen when there is concurrent involvement of the conductive airways. These images were obtained at the age of 3 years during initial episode of pneumonia.

**Figure 4:**
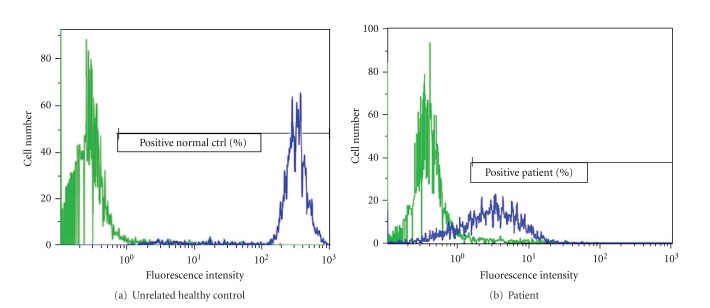
Dihydrorhodamine-123 (DHR) fluorescence assay in peripheral-blood neutrophils from the patient and an unrelated healthy control are displayed. In this assay, neutrophils are stimulated with phorbol myristate acetate (PMA) in the presence of DHR, and flow cytometry is used to measure the oxidative burst mediated by NADPH oxidase by quantifying the fluorescent product. After PMA stimulation, the histogram from the control (a) shows a unimodal shift of fluorescence far to the right, whereas the patient has a broad-based shift in fluorescence that is only partially shifted to the right compared with the healthy control (b).

**Figure 5:**
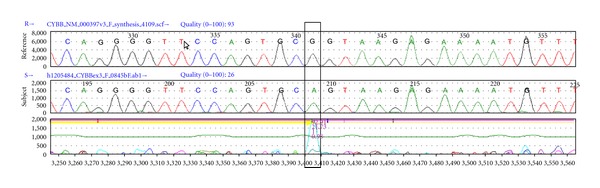
Genomic DNA sequence of the proband gp-91-phox transcript. Sanger sequencing of a normal CYBB gene (reference) and the patient (subject) showing c.252 G>A mutation of an alanine codon (GCG to GCA), followed by a classic splice donor signal GTAAG in the intron (lower panel).

Evaluations were undertaken to identify an underlying cause of recurrent infections limited to the lungs. Review of his current and previous chest images revealed the presence of focal opacities in different areas of the lung (Figures 2 and 3). The size and distribution of these opacities were variable, from extensive nodularity (Figure 2(a)) to large focal opacifications involving different segments and/or lobes of the lungs (Figures 2(b) and 2(c)). The diffuse and recurrent nature of his pulmonary lesions raised the concern for an underlying immunodeficiency. Further immunologic evaluations showed IgG levels of 1610 mg/dL and normal absolute lymphocyte count (ALC) with normal CD4/CD8 ratio; however, his neutrophil oxidative burst was abnormal according to dihydrorhodamine-123 (DHR) testing. Specifically, DHR test identified 34% of neutrophils negative for DHR fluorescence after phorbol myristate acetate (PMA) stimulation, with the remaining 66% of neutrophils being only dimly positive after stimulation (Figure 4). These DHR results suggested the diagnosis of CGD, which was confirmed later by detecting a mutation (c.252 G>A, p.Ala84Ala) in the CYBB gene (Figure 5).

The patient continued to be febrile and was without signs of improvement after three days of treatment with vancomycin and cefepime. Voriconazole and trimethoprim/sulfamethoxazole (TMP-SMX) were added, and the patient started to improve. He was discharged home to complete a four-week course of antibiotics. In addition, trimethoprim/sulfamethoxazole (TMP-SMX) and itraconazole were prescribed for long-term CGD prophylaxis. At follow-up visits, the patient has remained afebrile, and cough and respiratory findings have resolved.

## 3. Discussion

Not all CGD cases present with the same severity. In particular, the autosomal recessive forms of the disease may have a milder more insidious clinical course [9–11]. In contrast, patients with X-linked variants, which are due to mutations in the CYBB gene [1–3], tend to present early in life with multiple, sometimes fatal, infections resulting from complete absence of NADPH oxidase function [6–8]. To our knowledge, this is the first reported case in the literature of a patient with a CYBB gene mutation (c.252 G>A, p.Ala84Ala) presenting only isolated lung involvement. The phenotypic picture of this patient was remarkable because, despite having an X-linked mutation, he had residual neutrophil activation after stimulation, a pattern typically seen in autosomal recessive variants, where the ability of phagocytes to generate superoxide anion is only partially impaired [9–11]. As a result, this child had an atypical mild form of X-linked CGD remaining undiagnosed for several years.

Prior reports have identified atypical presentations and adult forms of X-linked CGD resulting from rare CYBB mutations with residual NADPH oxidase. For instance, Brunner et al. reported an X-linked CGD case with partially preserved oxidase activity and sarcoidosis-like picture caused by an intraexonic splice defect in the gene encoding gp91-phox (CYBB exon 3, c.262G->A) [12]. Another X-linked case with residual NADPH oxidase activity (in-frame triplet deletion in gp91-phox gene) was seen in an adult with multisystem disease that included staphylococcal lymphadenitis, recurrent pneumonia, and liver/renal abscesses [13]. Similarly, a gp91-phox gene splice site mutation (5′intron3 GTAAG/GTAAA), with residual NADPH, was described in a 40-year-old man with liver abscesses (*Staphylococcus aureus*) and septicemia (*Salmonella enteritis*) [14]. More severe phenotypes have also been reported in adults with point mutations in the CYBB gene's promoter (insertion of a T at position −54T to −56T), despite the presence of residual NADPH oxidase activity [15]. Collectively, these clinical reports demonstrate that some CYBB mutations induce only partial loss of NAPDH function, which results in less severe phenotypes; however, this residual NAPDH oxidase activity is not sufficient to completely protect the patient against infections.

Independent of the severity, CGD is typically a condition characterized by multisystem compromise [1–5]. Although pneumonia occurs in about 80% of CGD cases [16], infections in other systems, such as skin, lymph nodes, or bones, are commonly present in this disease [1–3]. In contrast, recurrent pneumonia was the only manifestation of CGD in our case. Because the differential diagnosis of such isolated lung involvement is broad, it is important to identify the pulmonary radiographic features suggestive of CGD that may allow timely diagnosis of this condition. In this regard, prior literature has described typical radiological patterns of granulomatous inflammation and infection in the lung of patients with CGD [17, 18], which include focal consolidation, abscess formation, reticulonodular opacities, and diffuse miliary infiltration [17, 18]. Lymphadenopathy, pulmonary fibrosis, pleural thickening, and contiguous extension of disease from the lungs to the pleura or chest wall (i.e., ribs or vertebral osteomyelitis) have also been reported in patients with CGD, particularly in those cases with infection by *Aspergillus* [18]. Similarly, our patient had focal opacities with variable patterns, ranging from extensive diffuse nodularity (Figures 2(a) and 3) to large opacifications involving different segments and/or lobes of the lungs (Figures 1 and 2). There were essentially absence of hyperinflation and other signs of small airway disease in his chest radiographs and CT scan. Collectively, these radiographic images raised the concern for an underlying immunodeficiency, particularly a phagocytic defect like CGD, that could lead to isolated and recurrent lung abscesses and/or granulomas without significant compromise of the conductive airways.

Another clinical feature of importance when considering the diagnosis of CGD is the lack of response to standard antibiotic therapy during bacterial respiratory infections. This is because the underlying phagocytic defect results in vulnerability to uncommon microorganisms. Specifically, recurrent infections in patients with CGD are typically due to *Staphylococcus aureus, Serratia marcescens, Burkholderia cepacia, Nocardia,* and *Aspergillus* species [1, 2]. Not surprisingly, our patient had a presumptive *Aspergillus* lung infection, with positive titers in BAL fluid (Galactomannan testing) and a significant clinical response to antifungal therapy with voriconazole. However, *Streptococcus viridans*, which is inherently a catalase negative bacteria, was isolated in BAL cultures (>100,000 colonies). In this regard, it is noteworthy that *Streptococcus* species are a well-known cause of disease in neutropenic hosts [19] and have recently been recognized as important pathogens in CGD [20]. Indeed, streptococcal infections caused by *S. intermedius*, *S. mitis*, or *S. anginosus* may lead to pyogenic liver abscesses in individuals with CGD [20]. Moreover, *S. viridans* have also been reported to cause mastoiditis, periauricular abscesses, and cervical adenitis in patients diagnosed with autosomal recessive variants of CGD [21, 22]. To our knowledge, our case is the first report of a pulmonary infection caused by *S. viridans* in a patient with X-linked CGD.

The X-linked CGD-causing mutation identified in our patient was c.252 G>A, p.Ala84Ala, a mutation in the exon 3 of CYBB gene that affects splicing and lowers the expression of gp91-phox [6]. The c.252 G>A mutation has been previously described using alternative nomenclature as c.264 G>A [6]. This nucleotide substitution results in the replacement of codon Ala84 from GCG to GCA, both of which code for an alanine residue. Although this mutation does not change the predicted amino acid at this position, it prevents splicing of exon 3 to exon 4, causing exon 3 to be deleted in the patient's mRNA [11]. It is proposed that the amino acid sequence encoded in exon 3 is essential for forming a necessary local tertiary structure in the first intracellular domain for p47phox binding and p22 interaction. Absence of this interaction seems to be detrimental to the stability of the enzyme complex [11]. This mutation has been previously reported in late onset cases of CGD with residual reactive oxygen species (ROS) production [7], although complete absence of NADPH oxidase activity has also been described [8, 11]. The mechanisms for which this phenotypic variability occurs are still unclear. It is possible that other genetic loci and/or other factors (i.e., epigenetic and environmental) may be implicated [11, 23]. Future research is required to conduct systematic correlations of clinical outcome, NAPDH functional data, and genetic mutations in order to better characterize specific subgroups of patients with CGD.

In summary, the clinical diagnosis of the mild forms of X-linked CGD may be extremely challenging and requires a high index of suspicion. For pediatric clinicians, information on the causative organism, severity, and location of the episodes, as well as pulmonary radiographic findings present, can provide critical diagnostic clues. Given that early therapy can improve survival in CGD, the prompt identification of atypical X-linked CGD variants with residual NADPH oxidase, in cases such as our patient, can significantly impact the clinical outcome of children with this condition.

## Abbreviations

CGD: Chronic granulomatous disease
NADPH: Nicotinamide adenine dinucleotide phosphate
CYBB: Cytochrome b-245 beta
DHR: Dihydrorhodamine-123.
